# Experimental study of superheating of tin powders

**DOI:** 10.1038/s41598-020-76223-x

**Published:** 2020-11-04

**Authors:** Han Gil Na, Youngmin Byoun, Suyoung Park, Myung Sik Choi, Changhyun Jin

**Affiliations:** 1 UDerive, Business R&D Center 605, Inharo 100, Nam-gu, Inha University, Incheon, 22212 Republic of Korea; 2 Metal & Machinery Team, Korea Conformity Laboratories (KCL), Seoul, 08503 Republic of Korea; 3 Department of Semiconductor Materials and Applications, Korea Polytechnic, 398 Sujeong‑ro, Sujeong‑gu, Seongnam‑si, Gyeonggi‑do 13122 Republic of Korea; 4 Department of Materials Science and Engineering, Yonsei University, Seoul, 03722 Republic of Korea

**Keywords:** Chemistry, Materials science

## Abstract

An unstable energy-unbalanced state such as superheating or supercooling is often unexpectedly observed because a factor of energy depends not only on the temperature but is a product of temperature (T) and entropy (S). Thus, at the same temperature, if the entropy is different, the total energy of the system can be different. In such cases, the temperature-change-rate cannot match the entropy-change-rate, which results in a hysteresis curve for the temperature/entropy relationship. Due to the difference between the temperature- and entropy-change-rates, properties of a material, such as the boiling and freezing points, can be extended from point to area. This study confirmed that depending on the heating rate, tin powders exhibit different melting points. Given the contemporary reinterpretation of many energy-non-equilibrium phenomena that have only been discussed on the basis of temperature, this study is expected to contribute to the actual expansion of scientific/engineering applications.

## Introduction

All physicochemical reactions attempt to maintain energy equilibrium; however, phenomena in the energy non-equilibrium state are frequently observed in everyday life. Representative examples are superheating^1,2^ and supercooling^3,4^. First, in the dictionary sense, superheating means that the solution contains a greater amount of solute than the solubility at any specified temperature, and supercooling means cooling a liquid below its freezing point without solidification/crystallisation. Superheating and supercooling both represent energy non-equilibrium states; however, the heating and cooling times can be adjusted slowly and rapidly, respectively; to create a desired unstable state. In other words, the opposite velocity concepts of ‘slow heating time’ and ‘fast cooling time’ are involved in this reaction. Due to this concept of time-related physical quantities (i.e. heating rate or cooling rate), it is possible to form a new material property zone from a zero-dimensional point (or a one-dimensional line), such as the boiling^5,6^ and freezing points^7,8^, to a two-dimensional area (or a three-dimensional volume). Thus, heating or cooling rates are factors that can control the dimensions that represent material properties because they can freely transcend the boundaries of dimensions through the time factor.

Nevertheless, it is easy to intuitively mistake the change in temperature to be the change in energy because entropy increases with temperature^9^. Therefore, energy expressed as the product of temperature and entropy can be mistaken to be increasing as the temperature increases, while ignoring the effect of entropy. However, it is important to note that the temperature-change-rate is not directly proportional to the entropy-change-rate. Therefore, there may be cases where the temperature-change-rate may match the entropy-change-rate, or may be faster or slower. Therefore, a hysteresis curve relationship can be established due to the mismatch between the temperature- and the entropy-change-rates of the material^10–12^.

In this study, to observe the difference (change) in the reaction over time, we investigated the possibility for supersaturation, saturation, and unsaturation in the temperature-solubility curve basis the different heating rates of tin powders. Moreover, under different cooling rates, which are opposite of the heating rates, a series of supercooling, nucleation, and isothermal solidification states in the time–temperature curve were deduced. Thus, unlike intuitive interpretation that relies only on temperature based on the heating or cooling rates of the material, the principle that two different phases, such as solid and liquid, can exist simultaneously was recognised in this study. In addition, we attempted to derive a new interpretation (cause) of the physicochemical phenomena (result) by introducing the factor of the heating rate, which we are familiar with at such a sequential moment change.

## Results and discussion

### Features in differential scanning calorimetry (DSC) experiments on tin powders

To analyse the experimental results and the causes of the melting point of tin powders that vary depending on the heating rate, it is necessary to first organise the experimental characteristics as thermodynamic data. Enthalpy (H) is the energy that can be drawn from the thermodynamic system and can be expressed as^13,14^

1 $$ {\text{H }} = {\text{ U }} + {\text{ PV}} $$

where H, U, P, and V indicate the enthalpy of the system, internal energy of the system, pressure of the system, and volume of the system, respectively. At this stage, as in our experiment, if the temperature is increased at different rates but there is no change in the pressure ($\Delta$P = 0), then the change (${\Delta}$) in enthalpy can be expressed as the difference between the heat exchanged inside and outside the system^13,14.^

2 $$ \Delta{\text{H }} = \Delta{\text{ U }} + {\text{P}}\Delta{\text{V}} + {\text{V}}\Delta{\text{P}}=\Delta{\text{ U }}+{\text{P}}\Delta{\text{V}} =\Delta{\text{Q}} $$

where Q is calories. Therefore,

3 $$ {\text{dQ }} = {\text{ dU }} + {\text{ PdV}} $$

where d is a small difference. However, since our tin powders correspond to a solid, we considered a negligible volume change even in the DSC measurement (dV ≈ 0). Thus, dQ can be expressed as.

4 $$ {\text{dQ}}\, = \,{\text{dU}} $$

In other words, the change in internal energy can all appear as a change in calories. However, according to the thermodynamic definition, dQ may be expressed as TdS and dU may be expressed as cmdT^15,16^: where T, S, c, and m indicate the temperature of the system, entropy of the system, specific heat capacity of the system, and mass of the system, respectively.

At this stage, the values of “c” and “m” remained unchanged, regardless of whether the temperature of the tin powders was 25 or 300 °C. That is, since the pressure and volume before and after the reaction hardly change, even when treated as a molar heat capacity as c_p_ or c_v_, the result remained unaffected: where c_p_ and c_v_ are molar heat capacity under constant pressure and constant volume, respectively^15,16^.

From the relationship above,

5 $$ {\text{TdS }} = {\text{ c}}_{{\text{p}}} {\text{dT}} $$

6 $$ {\text{dS }} = {\text{c}}_{{\text{p}}} {\text{dT}} $$

**Figure 1:**
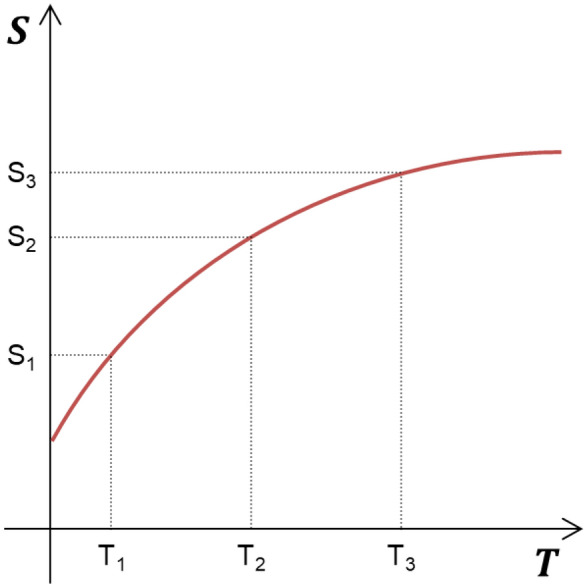
Typical changes in molar entropy with temperature in elements or compounds.

When Eq. (6) is integrated as follows,

7 $$ \int\limits_{{{\text{S}}_{1} }}^{{{\text{S}}_{2} }} {{\text{ds}}}  = \int\limits_{{{\text{T}}_{1} }}^{{{\text{T}}_{2} }} {\frac{1}{{\text{T}}}} {\text{c}}_{{\text{p}}} {\text{dT}}$$

a graph of temperature-entropy of S_2_ − S_1_ = c_p_. ln $\frac{{\text{T}}_{2}}{{\text{T}}_{1}}$ can be obtained as shown in Fig. 1.

### Change of melting point of tin powders according to different heating rates and different amount of tin powder

**Figure 2:**
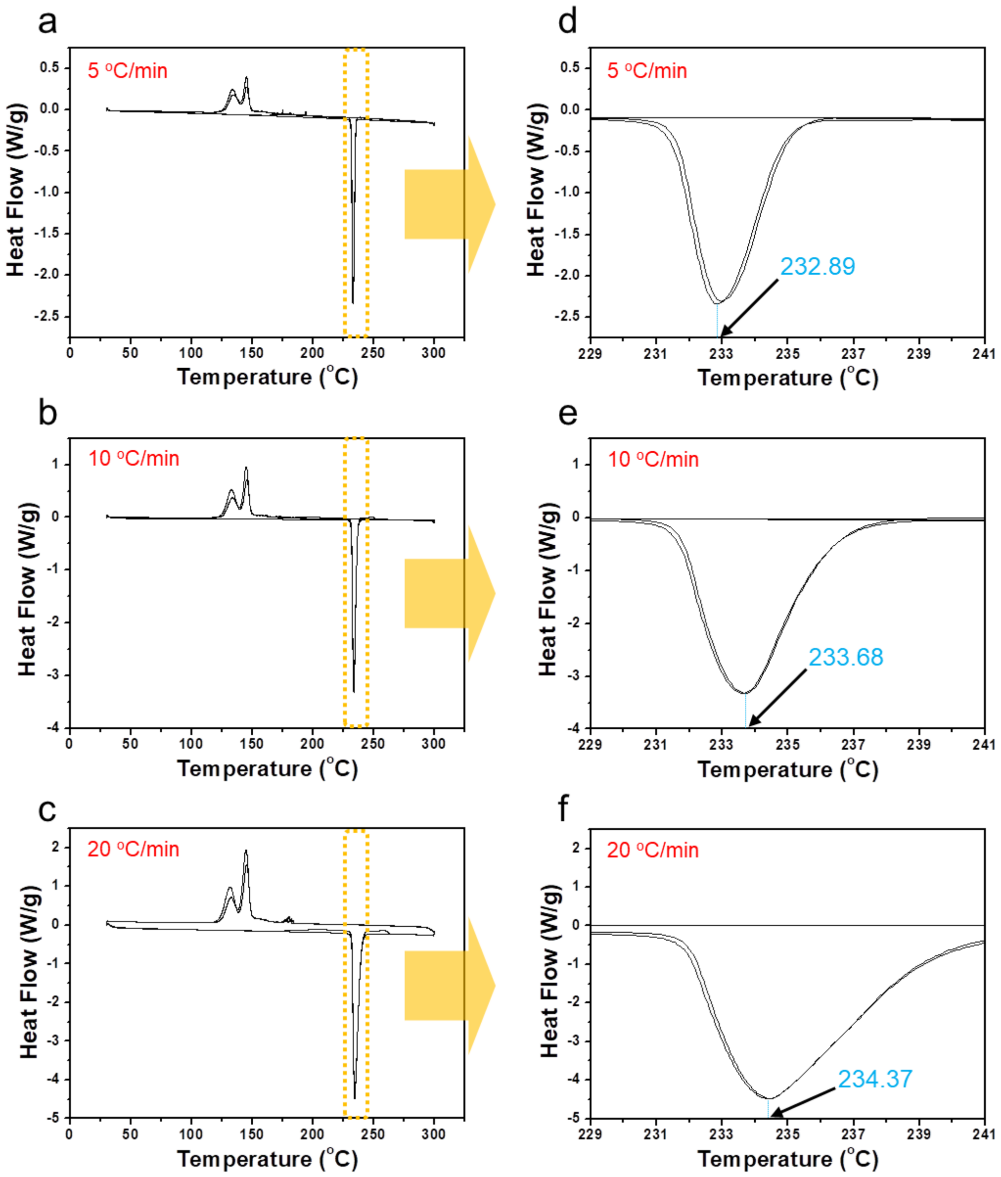
DSC analysis in the same 9 mg tin powders with different heating rates. DSC graphs with (**a**,**d**) the heating rate of 5 °C/min; (**b**,**e**) the heating rate of 10 °C/min; (**c**,**f**) the heating rate of 20 °C/min. Then, even in the case of tin powders having the same heat capacity and mass, the melting point of the tin powders may vary depending on the heating rate.

The results show that the energy and entropy for a single material can be changed if heat and amount of tin powder are applied differently. First, after preparing some tin powders with a predetermined melting point^17^, we try to change the phase by melting the tin powders at three different heating rates (i.e., 5 °C/min, 10 °C/min, 20 °C/min) and three different amount of tin powder (i.e., 5 mg, 9 mg, 12 mg). Figures 2a–f reveal the cases where the 9 mg tin powders were melted in the DSC analyser at heating rates of 5 (Fig. 2a,d), 10 (Fig. 2b,e), and 20 °C/min (Fig. 2c,f), respectively. The tin powders themselves are the same in terms of heat capacity^18^ and mass for the three different processes. First, Fig. 2a,d, which correspond to a heating rate of 5 °C/min, show a significantly lower melting point (232.89 °C in Fig. 2d) than that of the other samples (Fig. 2b,c,e,f). Second, Fig. 2b,e, which correspond to a heating rate of 10 °C/min, show a higher melting point (233.68 °C in Fig. 2e) than that in Fig. 2d. Third, Fig. 2c,f, which correspond to a heating rate of 20 °C/min, show the highest melting point (234.37 °C in Fig. 2f) among the three samples. It should be noted here that at different heating rates, tin powders have different melting points. The total energy (Q) for the melting of tin powders can be derived from Q = TS. Therefore, it can be predicted that a difference in the temperature of melting points according to the heating rate leads to a difference in the entropy (S) value, maintaining the total energy. According to the experimental results, even if the energy required to melt the tin powders is the same, the rate at which the temperature and the entropy contribute may vary depending on the heating rate, because if only the product of the two terms (temperature and entropy) is the same, the total energy remains the same. On the other hand, even when the amount of tin powder was decreased (5 mg, (Supplementary Information (SI), Fig. S1)) or increased (12 mg, (SI, Fig. S2)), as shown in Fig. 2, the melting points of tin powders at different heating rates (i.e., 5 °C/min, 10 °C/min, 20 °C/min) were different. Therefore, it can be seen that even if the amount of tin powder is changed (i.e., 5 mg, 9 mg, 12 mg), this phenomenological trend is maintained. That is, the possibility of hysteresis between two factors remains because there is a difference in temperature-change-rate and entropy-change-rate. So even in case of a rapid heating rate (or when heating is accelerated), a hysteresis relationship may form due to the mismatch of the temperature- and entropy-change-rates^10–12,19,20^. We strongly suspect that this speculation may result in an energy imbalance phenomenon such as superheating and supercooling.

### Temperature-entropy relationship to be considered when including the concept of heating velocity and heating acceleration

**Figure 3:**
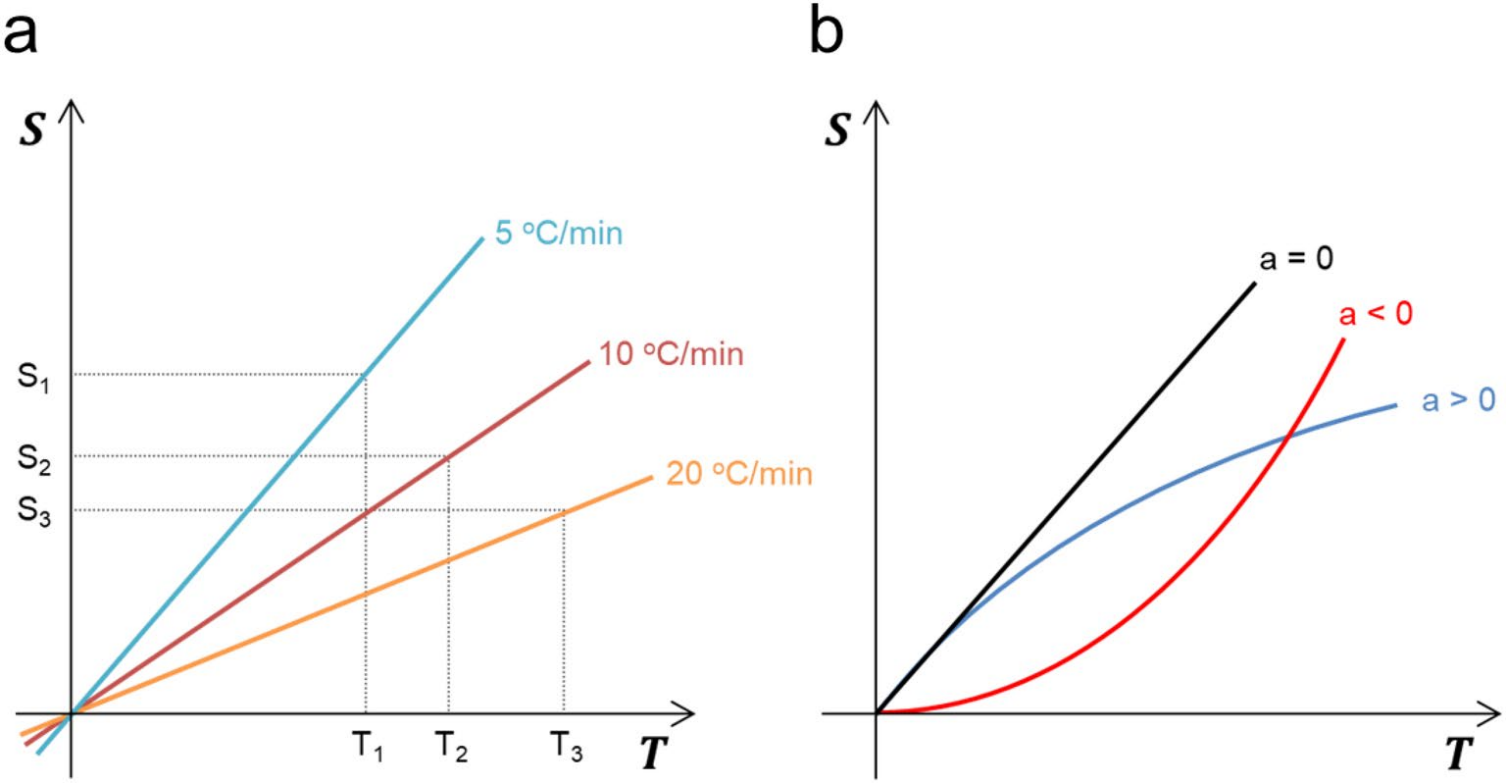
Schematic temperature-entropy relationship including the concept of velocity and acceleration. (**a**) Entropy and energy change according to the heating rate considered in a single dimension. (**b**) Temperature-entropy relationship according to acceleration, constant velocity, and deceleration.

For easy understanding of the experimental results, the graph of temperature-entropy, as shown in Fig. 1, is considered as a more outlined single dimension shown in Fig. 3. In this study, when the tin powders were melted at different heating rates, energy of at least a minimum melting point was required for the solid tin powders to melt. That is, the critical energy corresponding to the product of temperature and entropy was determined to melt the tin powders. Thus, in any way, if these energy values are achieved, tin powders can reach the melting point. From the experimental results in Fig. 2, we can predict that the rate of temperature increase affects the graph slope of temperature-entropy. That is, as shown in Fig. 3a, at three different heating rates, the temperature-entropy slope becomes smaller as the heating rate increases from 5 to 20 °C/min. The critical energy required to melt the tin powders can be expressed as the area of temperature and entropy. However, since each slope varies as the heating rate increases, the melting temperature of tin powders gradually increases as shown in Fig. 2, but the entropy value gradually decreases. That is, even at different heating rates, the product of temperature and entropy can always be maintained. The above case demonstrates the results when there is no acceleration or deceleration of the heating rate (a = 0). However, we must also consider the effects of heating acceleration (a > 0) or heating deceleration (a < 0) on the experiment. According to the results shown in Fig. 2, as the heating rate increases, the slope of the temperature-entropy curve gradually decreases. Therefore, when there is heating acceleration, the slope gradually decreases (Fig. 3b). Conversely, when there is a heating deceleration, the slope gradually increases as shown in Fig. 3b. Based on this principle, the melting point can be changed, and it is considered that an energy imbalance phenomenon can be artificially formed when the heating rate is extremely fast or extremely slow.

### Trend of actual temperature-entropy graph according to heating rate

**Figure 4:**
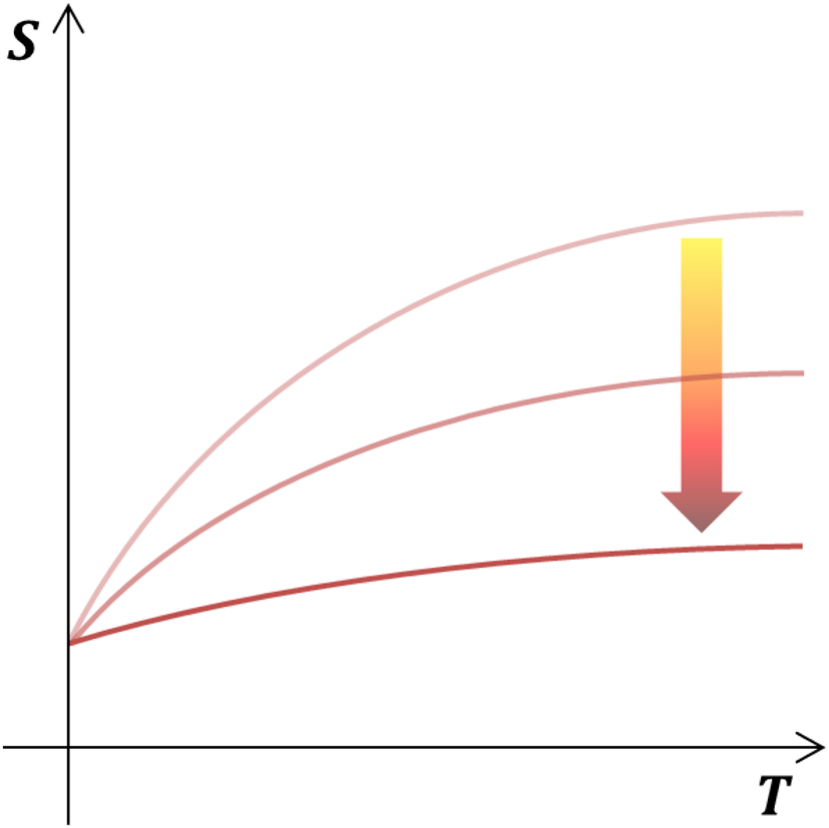
In the actual temperature-entropy graph, the trend of the graph changes according to the increase in heating rate.

The above case is the simplest approach to understand. Applying to the actual temperature-entropy relationship, the approach can be represented as shown in Fig. 4. That is, when the heating rate is increased or accelerated, the slope of the curve gradually decreases. However, if it is further expanded, the slope of the curve can increase over the straight line as seen in a < 0 in Fig. 3b. In our experiments, we did not directly obtain the results for various heating rates and accelerations. Nevertheless, the possibility of hysteresis of temperature and entropy when the temperature change increases can be inferred by the change in the melting points of tin powders. We estimated that the temperature difference between the inside and outside of the system acted as a driving force that resulted in this hysteresis phenomenon. As described above, we suggested only a simple concept that the final energy can be changed as the heating rate of tin changes. The concepts derived from these simple experiments may seem incomplete, but they contain sufficient reason to continue development. In addition, this approach could certainly help in a more systematic interpretation of more complicated heat transfers^21^.

## Conclusion

By controlling the dynamic conditions, such as the heating rate, the properties of the material can be extended to the concepts of area and volume rather than points and lines because energy is not expressed only in terms of the temperature but as a product of temperature and entropy. We confirmed this basis the fact that tin powders have differing melting points at different heating rates. That is, the rate of temperature increase may act as a cause of the hysteresis phenomenon between temperature and entropy. Therefore, even in other physicochemical phenomena related to temperature, efforts to find and solve many different phenomena that can be accessed as TS terms, compared with the existing T term only, will continue.

## Methods

**Figure 5:**
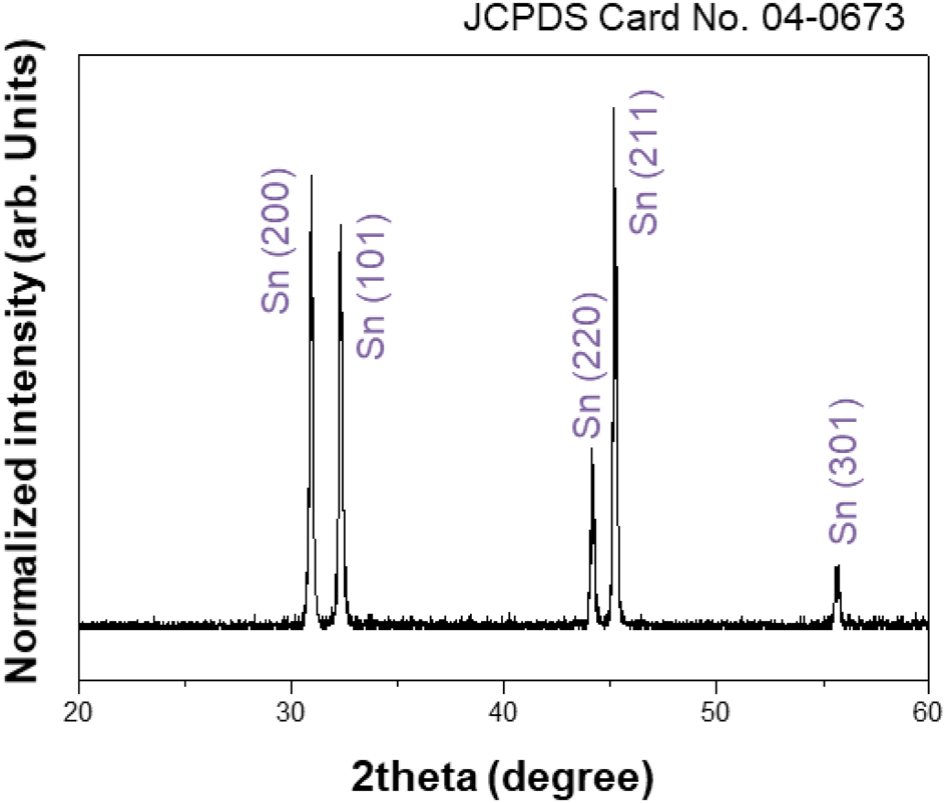
XRD indicating that the raw material is a tin powder.

To determine the relationship between temperature, entropy, and energy of 5 mg, 9 mg, 12 mg of tin (Sn) powders (Fig. 5) with different heating rates (i.e. 5, 10, 20 °C/min), the temperature-sample weight results were obtained using thermogravimetric analysis (Model: SDT Q600, TA Instruments) at temperatures ranging from 25 to 300 °C. Because of the difference in the entropy-change-rate at the same temperature difference (275 °C), depending on the heating rate, it is possible to analyse the different energy states different from the equilibrium energy.

## Supplementary information

Supplementary Information.

## Data Availability

All the data are available from the corresponding author on reasonable request.
