# Supplementary material for: Experimental study of superheating of tin powders
Source: Sci Rep. 2020 Nov 4;10:19026. doi: 10.1038/s41598-020-76223-x (PMC7643175; doi:10.1038/s41598-020-76223-x)
Supplement: Supplementary file 1 — Supplementary Information. [file 41598_2020_76223_MOESM1_ESM.docx]

**Supplementary information**

**Experimental study of superheating of tin powders**

Han Gil Na^1^, Youngmin Byoun^2^, Suyoung Park^3^, Myung Sik Choi^4^* & Changhyun Jin^4^*

^1^UDerive, Business R&D Center 605, Inharo 100, Nam-gu, Inha University, Incheon, 22212, Republic of Korea

^2^Metal & Machinery Team, Korea Conformity Laboratories (KCL), Seoul 08503, Republic of Korea

^3^Department of Semiconductor Materials and Applications, Korea Polytechnic, 398 Sujeong‑ro, Sujeong‑gu, Seongnam‑si, Gyeonggi‑do 13122, Republic of Korea

^4^Department of Materials Science and Engineering, Yonsei University, Seoul, 03722, Republic of Korea

Han Gil Na and Youngmin Byoun had equal contribution as co-first authors.

*Correspondence to: choigocms@yonsei.ac.kr (M.S.C), z8015026@yonsei.ac.kr (C. Jin)


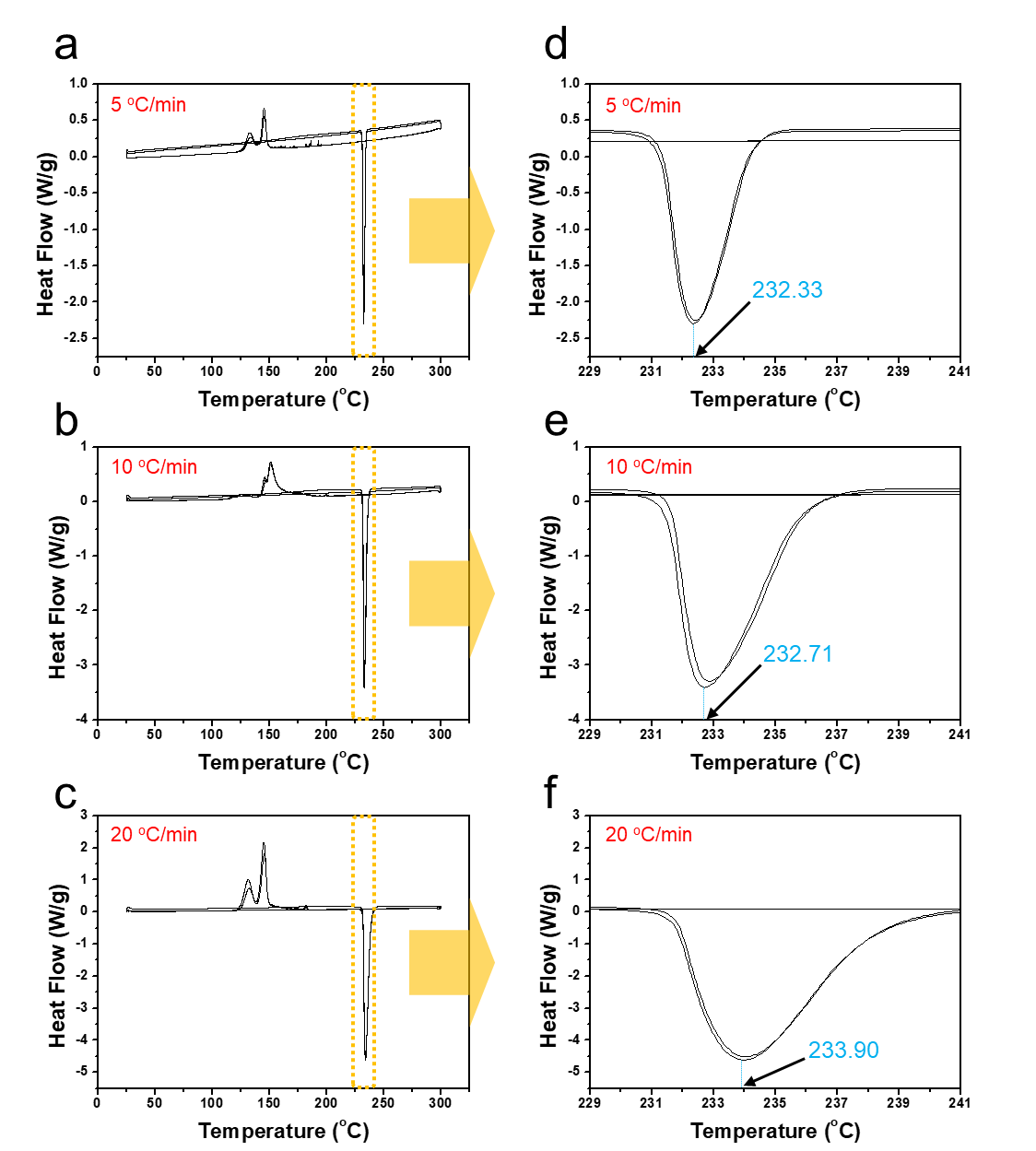


**Supplementary Figure S1 | DSC analysis in the same 5 mg tin powders with different heating rates.** DSC graphs with (a,d) the heating rate of 5 °C/min; (b,e) the heating rate of 10 °C/min; (c,f) the heating rate of 20 °C/min.


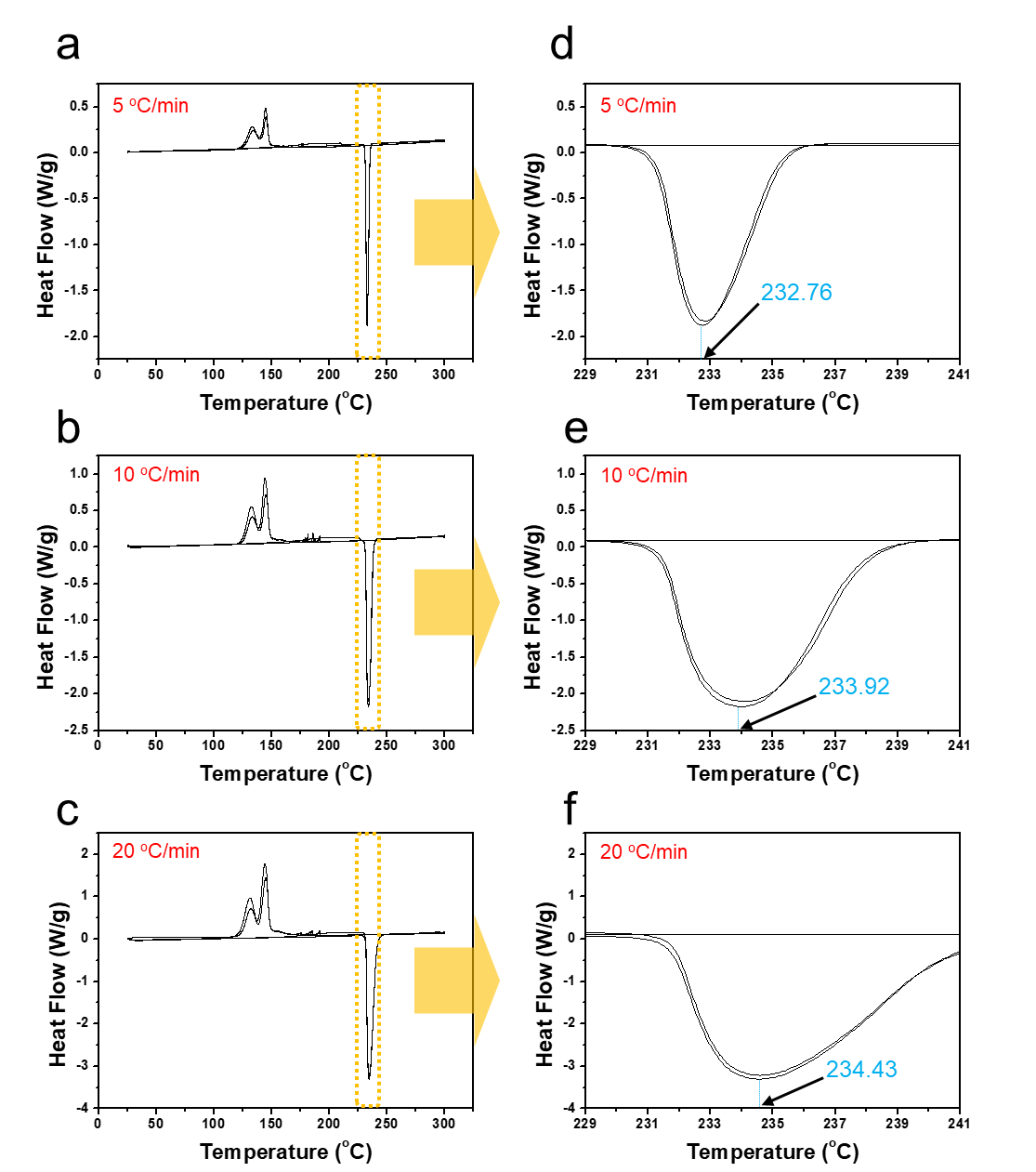


**Supplementary Figure S2 | DSC analysis in the same 12 mg tin powders with different heating rates.** DSC graphs with (a,d) the heating rate of 5 °C/min; (b,e) the heating rate of 10 °C/min; (c,f) the heating rate of 20 °C/min.
